# Supplementary material for: Simultaneous detection of omicron and other SARS-CoV-2 variants by multiplex PCR MassARRAY technology
Source: Sci Rep. 2023 Feb 6;13:2089. doi: 10.1038/s41598-023-28715-9 (PMC9900542; doi:10.1038/s41598-023-28715-9)
Supplement: Supplementary file 1 — Supplementary Tables. [file 41598_2023_28715_MOESM1_ESM.doc]

| **Supplement Table 1.** Mean % call rate of 5 virus isolates at 10 to 10,000 copies/uL tested with PMA-ABDO | | | | | | | | | |  |
| --- | --- | --- | --- | --- | --- | --- | --- | --- | --- | --- |
|  |  | Mean % call rate* | | | | |  |  |  | |
| Copies/ul | ln | Alpha | Beta | Delta | Omicron | Ancestral |  |  |  | |
| 100000 | 11.51293 | 100 | 96.88 | 96.88 | 100 | 100 |  |  |  | |
| 10000 | 9.21034 | 100 | 93.75 | 90.62 | 100 | 100 |  |  |  | |
| 1000 | 6.907755 | 96.88 | 93.75 | 81.25 | 100 | 96 |  |  |  | |
| 100 | 4.60517 | 81.25 | 84.38 | 81.25 | 92 | 92 |  |  |  | |
| 10 | 2.302585 | 71.88 | 62.5 | 81.25 | 76 | 76 |  |  |  | |

* The RNA samples were tested duplicate.

| **Supplement Table 2.** The PMA-ABDO detection limit for SAS-CoV-2 variant identification. RNA extract from 5 SARS-CoV-2 viral isolates (ancestral, Alpha, Beta, Delta, and Omicron) was serial dilution from 10,000 to 1 copies/uL | | | | | |
| --- | --- | --- | --- | --- | --- |
| Mutation site | Detection limit (copies/uL) [detected nucleotide by MassARAY] | | | | |
|  | Ancestral* | Alpha (B.1.1.7)* | Beta (B.1.351)* | Delta (B.1.617.2)* | Omicron (B.1.529)* |
| L18F | 10 [C] | 10 [C] | 10 [C] | 10 [C] | 10 [C] |
| T19R | 10 [C] | 10 [C] | 10 [C] | 10 [G] | 10 [C] |
| DEL69/70 | 100 [TACATG] | 10 [DEL] | 10 [TACATG] | 10 [TACATG] | 10[DEL] |
| D80A | 10 [A] | 10 [A] | 10 [C] | 10 [A] | 10 [A] |
| T95I | 10 [C] | 10 [C] | 10 [C] | 10 [C] | 10 [T] |
| G142D | 100 [G] | 100 [G] | 1000 [G] | 100 [A] | 100 [G] |
| DEL144/144 | 10 [TTA] | 10 [DEL] | 10 [TTA] | 10 [TTA] | 10 [TTA] |
| DEL157/158 | 10 (AGTTCA) | 10 (AGTTCA) | 10 (AGTTCA) | 10 (DEL) | 10 (AGTTCA) |
| D215G | 100 (A) | 10 (A) | 10 (G) | 10 (A) | 100 (A) |
| DEL241/243 | 10 (CTTTACTTG) | 10 (CTTTACTTG) | 10 (DEL) | 10 (CTTTACTTG) | 10 (CTTTACTTG) |
| K417N | 10 (G) | 10 (G) | 10 (T) | 10 (G) | 10 (T) |
| L452R | 10 (T) | 10 (T) | 10 (T) | 1000 (G) | 10 (T) |
| T478K | 10 (C) | 10 (C) | 10 (C) | 10 (A) | 10 (C) |
| E484A | 100 (A) | 10 (A) | 10 (A) | 10 (A) | 100 (C) |
| E484Q | 10 (G) | 10 (A) | 10 (A) | 10 (G) | 10 (G) |
| N501Y | 10 (A) | 10 (T) | 10 (T) | 10 (A) | 10 (T) |
| A570D | 10 (C) | 10 (A) | 10 (C) | 10 (C) | 10 (C) |
| D614G | 10 (A) | 10 (G) | 10 (G) | 10 (G) | 10 (G) |
| P681R/H | 1000 (C) | 1000 (A) | 1000 (C) | 1000 (G) | 1000 (A) |
| A701V | 10 (C) | 100 (C) | 10 (T) | 10 (C) | 10 (C) |
| T716I | 10 (C) | 10 (T) | 10 (C) | 10 (C) | 10 (C) |
| D950N | 10 (G) | 100 (G) | 10 (G) | 10 (A) | 10 (G) |
| S982A | 10 (T) | 10 (G) | 10 (T) | 10 (T) | 10 (T) |
| D1118H | 10 (G) | 10 (C) | 10 (G) | 10 (G) | 10 (G) |
| N gene | 10 (C) | 10 (C) | 10 (C) | 10 (C) | 10 (C) |

*GISAID accession ID: ancestral lineage (EPI_ISL_412028); Alpha variant (EPI_ISL_683466); Beta variant (EPI_ISL_678570); Delta variant (EPI_ISL_2510689); Omicron variant (EPI_ISL_14175998)

| **Supplement Table 3.** The PMA-Omicron detection limit for SAS-CoV-2 variant identification. RNA extract from 5 SARS-CoV-2 viral isolates (ancestral, Alpha, Beta, Delta, and Omicron) was serial dilution from 10,000 to 1 copies/uL | | | | | | |
| --- | --- | --- | --- | --- | --- | --- |
| Mutation site | Detection limit (copies/uL) [detected nucleotide by MassARAY] | | | | |  |
|  | Ancestral* | Alpha (B.1.1.7)* | Beta (B.1.351)* | Delta (B.1.617.2)* | Omicron (B.1.529)* |  |
| T19R | 10 (C) | 10 (C) | 10 (C) | 10 (G) | 10 (C) |  |
| A67V | 10 (C) | 10 (C) | 10 (C) | 10 (C) | 10 (T) |  |
| DEL69/70 | 10 (TACATG) | 10 (DEL) | 10 (TACATG) | 10 (TACATG) | 10 (DEL) |  |
| T95I | 10 (C) | 10 (C) | 10 (C) | 10 (C) | 10 (T) |  |
| DEL143/145 | 10 (C) | 100 (C) | 1000 (C) | 100 (C) | 100 (T) |  |
| DEL212/212 | 100 (ATT) | 10 (ATT) | 10 (ATT) | 10 (ATT) | 10 (DEL) |  |
| G339D | 10 (GTGTTTATT) | 10 (GTGTTTATT) | 10 (GTGTTTATT) | 10 (GTGTTTATT) | 10 (DEL) |  |
| R346K | 10 (G) | 10 (G) | 100 (G) | 1000 (G) | 10 (G) |  |
| S373P | 1000 (T) | 10 (T) | 10 (T) | 10 (T) | 10 (C) |  |
| S375F | 10 (C) | 10 (C) | 10 (C) | 10 (C) | 10 (T) |  |
| K417N | 10 (G) | 10 (G) | 10 (T) | 10 (G) | 10 (T) |  |
| S477N | 10 (G) | 10 (G) | 10 (G) | 10 (G) | 10 (A) |  |
| T478K | 10 (C) | 10 (C) | 10 (C) | 10 (A) | 10 (A) |  |
| E484A | 100 (A) | 10 (A) | 10 (A) | 10 (A) | 10 (C) |  |
| Q493R | 10 (A) | 10 (A) | 10 (A) | 100 (A) | 10 (G) |  |
| Q498R | 100 (A) | 10 (A) | 10 (A) | 10 (A) | 10 (G) |  |
| N501Y | 10 (A) | 10 (T) | 10 (T) | 10 (A) | 10 (T) |  |
| Y505H | 100 (T) | 10 (T) | 10 (T) | 1000 (T) | 10 (C) |  |
| T547K | 10 (C) | 10 (C) | 10 (C) | 100 (C) | 10 (A) |  |
| D614G | 10 (A) | 10 (G) | 10 (G) | 10 (G) | 10 (G) |  |
| H655Y | 10 (C) | 10 (C) | 10 (C) | 10 (C) | 10 (T) |  |
| P681R/H | 1000 (A) | 10 (A) | 10 (C) | 10 (G) | 10 (A) |  |
| D796Y | 10 (G) | 10 (G) | 10 (G) | 10 (G) | 10 (T) |  |
| N856K | 10 (C) | 10 (C) | 10 (C) | 10 (C) | 10 (A) |  |
| Q954H | 10 (A) | 10 (A) | 10 (A) | 10 (A) | 10 (T) |  |
| N969K | 10 (T) | 10 (T) | 10 (T) | 10 (T) | 10 (A) |  |
| L981F | 10 (C) | 10 (C) | 10 (C) | 10 (C) | 10 (T) |  |
| D1118H | 10 (G) | 10 (C) | 10 (G) | 10 (G) | 10 (G) |  |
| N gene | 10 (C) | 10 (C) | 10 (C) | 10 (C) | 10 (C) |  |

*GISAID accession ID: ancestral lineage (EPI_ISL_412028); Alpha variant (EPI_ISL_683466); Beta variant (EPI_ISL_678570); Delta variant (EPI_ISL_2510689); Omicron variant (EPI_ISL_14175998)

| Supplement Table 4. Detection results of target sites from nasopharyngeal swab samples in non-COVID-19 patients by PMA-ABDO (Total number =40) | | | | | | | | | | | | | | | | | | | | | | | | | | | | |
| --- | --- | --- | --- | --- | --- | --- | --- | --- | --- | --- | --- | --- | --- | --- | --- | --- | --- | --- | --- | --- | --- | --- | --- | --- | --- | --- | --- | --- |
| Sample No. | Type of Sample | L18F | T19R | DEL69/70 | D80A | T95I | G142D | DL144/144 | DEL157/158 | D215G | DEL241/243 | K417N | L452R | T478K | E484A | E484K_Q | N501Y | A570D | D614G | P681R/H | A701V | T716I | D950N | S982A | D1118H | N gene | Call-rate (%) |  |
| 1 | AdV | nc | nc | nc | A | nc | nc | nc | nc | nc | nc | nc | nc | nc | nc | nc | nc | nc | G | nc | nc | nc | nc | nc | nc | nc | 8 |  |
| 2 | Flu A/H1N1 | nc | nc | nc | nc | nc | nc | nc | nc | nc | nc | nc | nc | nc | nc | nc | nc | nc | nc | nc | nc | nc | nc | nc | nc | nc | 0 |  |
| 3 | Flu A/H1N1 | nc | nc | nc | nc | nc | nc | nc | nc | nc | nc | nc | nc | nc | nc | nc | nc | nc | nc | nc | nc | nc | nc | nc | nc | nc | 0 |  |
| 4 | Flu A /H3 | nc | nc | nc | A | nc | nc | nc | nc | nc | nc | nc | nc | nc | nc | nc | nc | nc | nc | nc | nc | nc | nc | nc | nc | nc | 4 |  |
| 5 | Flu A/H3 | nc | nc | nc | A | nc | nc | nc | nc | nc | nc | nc | nc | nc | nc | nc | nc | nc | nc | nc | nc | nc | nc | nc | nc | nc | 4 |  |
| 6 | Flu B | nc | nc | nc | A | nc | nc | nc | nc | nc | nc | nc | nc | nc | nc | nc | nc | C | nc | nc | nc | nc | nc | nc | nc | nc | 8 |  |
| 7 | Flu B | nc | nc | nc | A | nc | nc | nc | nc | nc | nc | nc | nc | nc | nc | nc | nc | C | nc | nc | nc | nc | nc | nc | nc | nc | 8 |  |
| 8 | EV71 | nc | nc | nc | A | nc | nc | nc | nc | nc | nc | nc | nc | nc | nc | nc | nc | nc | G | nc | nc | nc | nc | nc | nc | nc | 8 |  |
| 9 | CV-A16 | nc | nc | nc | nc | nc | nc | nc | nc | nc | nc | nc | nc | nc | nc | nc | nc | C | nc | nc | nc | nc | nc | nc | nc | nc | 4 |  |
| 10 | Hbov | nc | nc | nc | nc | nc | nc | nc | nc | nc | nc | nc | nc | nc | nc | nc | nc | C | nc | nc | nc | nc | nc | nc | nc | nc | 4 |  |
| 11 | HCoV 229E | nc | nc | nc | nc | nc | nc | nc | nc | nc | nc | nc | nc | nc | nc | nc | nc | A | nc | nc | nc | nc | nc | nc | nc | nc | 4 |  |
| 12 | HCoV OC43 | nc | nc | nc | nc | nc | nc | nc | nc | nc | nc | nc | nc | nc | nc | nc | nc | nc | nc | nc | nc | nc | nc | nc | nc | nc | 0 |  |
| 13 | HCoVOC43 | nc | nc | nc | A | nc | nc | nc | nc | nc | nc | nc | nc | nc | nc | nc | nc | nc | nc | nc | nc | nc | nc | nc | nc | nc | 8 |  |
| 14 | HCoV NL63 | nc | nc | nc | A | nc | nc | nc | nc | nc | nc | nc | nc | nc | nc | nc | nc | nc | nc | nc | nc | nc | nc | nc | nc | nc | 4 |  |
| 15 | HCoV HKU1 | nc | nc | nc | A | C | nc | nc | nc | nc | nc | nc | nc | nc | nc | nc | nc | nc | nc | nc | nc | nc | nc | nc | nc | nc | 8 |  |
| 16 | HPIV-1 | nc | nc | nc | A | nc | nc | nc | nc | nc | nc | nc | nc | nc | nc | nc | nc | nc | nc | nc | nc | nc | nc | nc | nc | nc | 4 |  |
| 17 | HPIV-2 | nc | nc | nc | nc | nc | nc | nc | nc | nc | nc | nc | nc | nc | nc | nc | nc | nc | nc | nc | nc | nc | nc | nc | nc | nc | 0 |  |
| 18 | HPIV-3 | nc | nc | nc | A | nc | nc | nc | nc | nc | nc | nc | nc | nc | nc | nc | nc | nc | nc | nc | nc | nc | nc | nc | nc | nc | 4 |  |
| 19 | HMPV | nc | nc | nc | A | C | nc | nc | nc | nc | nc | nc | nc | nc | nc | nc | nc | nc | nc | nc | nc | nc | nc | nc | nc | nc | 8 |  |
| 20 | RSV | nc | nc | nc | A | nc | nc | nc | nc | nc | nc | nc | nc | nc | nc | nc | nc | nc | nc | nc | nc | nc | nc | nc | nc | nc | 4 |  |
| 21 | Non COVID* | nc | nc | nc | nc | nc | nc | nc | nc | nc | nc | nc | nc | nc | nc | nc | nc | nc | nc | nc | nc | nc | nc | nc | nc | nc | 0 |  |
|  | FP | 0 | 0 | 0 | 13 | 2 | 0 | 0 | 0 | 0 | 0 | 0 | 0 | 0 | 0 | 0 | 0 | 5 | 2 | 0 | 0 | 0 | 0 | 0 | 0 | 0 |  |  |
|  | TN | 40 | 40 | 40 | 27 | 38 | 40 | 40 | 40 | 40 | 40 | 40 | 40 | 40 | 40 | 40 | 40 | 35 | 38 | 40 | 40 | 40 | 40 | 40 | 40 | 40 |  |  |
|  | % Specificity | 100 | 100 | 100 | 67.5 | 95 | 100 | 100 | 100 | 100 | 100 | 100 | 100 | 100 | 100 | 100 | 100 | 87.5 | 95 | 100 | 100 | 100 | 100 | 100 | 100 | 100 |  |  |

nc= no call-rate, AdV=Adenovirus, influenza=Flu, EV71=Enterovirus 71, CV-A16=Coxsackievirus A16, HBoV = Human bocavirus, HCoV= Human coronavirus, HPIV=Human parainfluenza viruses, HMPV=Human metapneumovirus, RSV= Respiratory Syncytial Virus, NON-COVID*= samples from SARS-CoV-2 PCR negative (n=20), FP=number of sample with false positive signal, TN= number of sample with true negative signal, % Specificity = TN / (FP + TN)x10
